# Bimanual reach to grasp movements after cervical spinal cord injury

**DOI:** 10.1371/journal.pone.0175457

**Published:** 2017-04-06

**Authors:** Laura Britten, Rachel Coats, Ronaldo Ichiyama, Wajid Raza, Firas Jamil, Sarah Astill

**Affiliations:** 1 School of Biomedical Sciences, Faculty of Biological Sciences, University of Leeds, Leeds, United Kingdom; 2 School of Psychology, Faculty of Medicine and Health, University of Leeds, Leeds, United Kingdom; 3 Yorkshire Regional Spinal Injuries Centre, Pinderfields General Hospital, Wakefield, United Kingdom; University of Toronto, CANADA

## Abstract

Injury to the cervical spinal cord results in bilateral deficits in arm/hand function reducing functional independence and quality of life. To date little research has been undertaken to investigate control strategies of arm/hand movements following cervical spinal cord injury (cSCI). This study aimed to investigate unimanual and bimanual coordination in patients with acute cSCI using 3D kinematic analysis as they performed naturalistic reach to grasp actions with one hand, or with both hands together (symmetrical task), and compare this to the movement patterns of uninjured younger and older adults. Eighteen adults with a cSCI (mean 61.61 years) with lesions at C4-C8, with an American Spinal Injury Association (ASIA) grade B to D and 16 uninjured younger adults (mean 23.68 years) and sixteen uninjured older adults (mean 70.92 years) were recruited. Participants with a cSCI produced reach-to-grasp actions which took longer, were slower, and had longer deceleration phases than uninjured participants. These differences were exacerbated during bimanual reach-to-grasp tasks. Maximal grasp aperture was no different between groups, but reached earlier by people with cSCI. Participants with a cSCI were less synchronous than younger and older adults but all groups used the deceleration phase for error correction to end the movement in a synchronous fashion. Overall, this study suggests that after cSCI a level of bimanual coordination is retained. While there seems to be a greater reliance on feedback to produce both the reach to grasp, we observed minimal disruption of the more impaired limb on the less impaired limb. This suggests that bimanual movements should be integrated into therapy.

## Introduction

Many activities of daily living require the two hands to interact simultaneously with each other to accomplish functional reach-to-grasp tasks [1]. Furthermore, bimanual actions require both intralimb (reach and grasp) and interlimb coordination i.e. the hands have to function together [2]. After a cervical spinal cord injury (cSCI), the cord is often damaged bilaterally [3] and this results in functional impairments in both limbs [4] [5]. For an individual with tetraplegia this means that they have to regain function not only of the arms and hands independently, but also the ability to move both limbs together in a functionally meaningful way. Despite this, and the growing trend for bimanual training strategies [6], there is limited information on bimanual control strategies after SCI and how the movement of one limb affects the other during bimanual tasks [5].

Unimanual reach-to-grasp actions have been examined extensively, and several landmark kinematic characteristics have been identified which aids our understanding of movement control [7], [8], [9]. Compared to uninjured participants (UP), the transport phase of a unimanual reach-to-grasp in individuals with a cSCI, is slower [10], longer in duration [11–13], with a longer deceleration phase; which is suggested to represent the need to use visual feedback to guide the hand to the object [11–14]. During grasping, maximal grasp aperture is scaled to object size [15], but performed earlier in the transport phase, resulting in the reach and grasp being performed sequentially [11].

In UP bimanual reach and grasp actions take longer to complete, have lower peak velocities, longer deceleration phases (suggesting the need for more visual feedback), and have larger grasp apertures than unimanual movements [16,17]. Furthermore, the movement of one arm affects temporal and spatial kinematics of the contralateral arm. Early research shows that UP produce temporally synchronous bimanual movements, irrespective of the distance each limb travels [16],[17]. However, more recent work argues that while the hands may start moving together (and end in temporal proximity), there is coordinative asynchrony during the movement, even when moving the same distance [18,19] which could be due to the need to look from one hand to the other [18–20].

Research that has examined bimanual reach-to-grasp actions and interlimb synchrony of the limbs during bimanual functional tasks after cSCI is limited to one study following chronic cSCI [5]. This study showed that the more impaired limb had a detrimental influence on the motion of the less impaired limb, in that the less impaired limb was excessively delayed during bimanual reach-to-grasp tasks. In addition, bimanual movements were longer, but grip aperture was scaled to object size. The authors also noted reduced interlimb synchrony between the time it took the hands to reach maximum grasp aperture and make contact with the object, which was exacerbated in those individuals who showed prolonged times to open the hand.

Despite the bilateral deficits in the chronic stage of injury [5] and the potential effectiveness of bimanual training strategies [6], to our knowledge there is little work which has examined bimanual reach-to-grasp actions in individuals with a cSCI in the acute stage of injury. In this stage of injury the spared sensorimotor pathways provide avenues to be exploited to facilitate functional improvement through task specific training [21] [22,23] [24]. Understanding bimanual control strategies at the acute stage of injury is crucial in further optimising rehabilitation strategies to aid recovery of arm and hand function, which for many individuals with a cSCI, is the most important goal during neuro-rehabilitation [25].

In this study, the objective was to use kinematic analyses to investigate bimanual control strategies after a cSCI. Based on previous work we expect that (1) individuals with a cSCI will exhibit spatially and temporally different kinematic parameters during reach-to-grasp actions compared to UP, (2) that bimanual reach-to-grasp tasks will be longer and slower than unimanual actions for all participants (cSCI and UP), and (3) participants with a cSCI will have greater asynchrony during the bimanual condition than UP.

## Methods

### Participants

Eighteen inpatients from two UK Spinal Injuries Centres, who were in the acute stage of recovery were recruited to participate (Participant characteristics are presented in Table 1). Persons with a cSCI were included if they were >18yrs old, could understand and follow verbal instructions, could give written consent and had no history of additional neurological impairment. The preferred limb of participants with an cSCI was determined as the less impaired limb, which was identified according to the Chedoke Arm and Hand Inventory-9 [26]. UP were recruited from the local community, and comprised 16 young adults (YA) (mean age = 23.68±4.54yrs; 14 right handed; 9 Female) and 16 older adults (OA) (mean age = 70.92± 7.2yrs; 12 right handed, 9 Female). We consider the recruitment of uninjured young (YA), and older (OA) participants necessary due to the changing demographic of cSCI [27], and the already well documented differences in control strategies between YA and OA during reach-to-grasp tasks [28,29]. Ethical approval was sought from the Biological Sciences Faculty Research Ethics Committee, University of Leeds and Leeds West Research Ethics Committee, NHS. All participants gave written informed consent and the procedures conformed to the declaration of Helsinki.

**Table 1:** Participants with a cSCI characteristics.

cSCI subject	Age (years)	Gender (M/F)	Level	ASIA	Aetiology	Time since injury (weeks)	More Affected limb	CAHAI-9
1	73	M	C6	B	NT	17	R	42
2	68	M	C5	D	T	7	L	60
3	67	M	C7	C	T	17	R	56
4	57	M	C8	C	NT	11	L	-
5	79	F	C5	D	T	23	L	62
6	69	M	C5	-	T	8	L	58
7	79	M	C5	C	NT	9	L	63
8	73	M	C4	C	T	18	R	52
9	65	M	C6	D	T	15	R	44
10	40	M	C6	D	T	14	L	63
11	65	M	C5	C	T	14	L	56
12	47	M	C5	D	T	29	L	63
13	56	M	C5	C	NT	21	L	-
14	45	M	C6	C	T	6	L	63
15	35	F	C5	D	NT	97	L	63
16	67	M	C4	D	T	6	L	63
17	86	M	C4	D	NT	10	L	63
18	37	M	C4	D	NT	7	L	63

cSCI: cervical Spinal Cord Injury; M: Male; F: Female; ASIA: American Spinal Injuries Association Classification; T: Traumatic; NT: Non-Traumatic; L: Left; R: Right.

### Experimental set-up

Participants sat in a chair or their wheelchair at a height adjustable table with the hips and knees at 90 degrees. Before testing, maximal active forward reach distance with the marker on the dorsum of the hand was recorded to standardize the object placement across participants with different arm lengths and disabilities.

We investigated the kinematics and coordination between the two hands while the participants were asked to reach and grasp (using a precision grip) either one (unimanual) or two (bimanual) plastic blocks (30mmX30mmx18mm), which were placed at 50% of each individual’s maximal reach, 20cm apart (10cm at either side of the participant’s midline), at a self-selected, comfortable speed. After task familiarisation, participants performed 24 trials; 8 trials with the preferred/less impaired limb (P/LI), 8 trials with the non-preferred/more impaired limb (NP/MI) and 8 bimanual trials (B), with the order of trials blocked and pseudo-randomised between participants. Participants were instructed to complete the task as fast and as smoothly as possible when ready following the go signal. All participants completed the required number of trials, without the need for additional trunk support or compensatory trunk movements. All participants had full view of the arms/hands and the objects during each trial.

### Data acquisition

**Fig 1:**
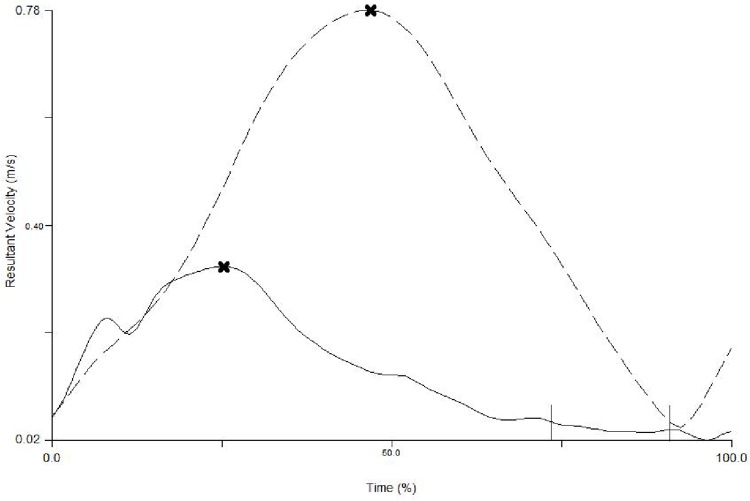
An example of a kinematic velocity profile for a participant with a cSCI (solid black line) and an uninjured young adult (dashed black line), in a unimanual condition (graphed between the start (0%) and the end of the movement (100%)) when the preferred/less impaired limb was reaching to the object. The cross markers show the average timing of peak velocity and the vertical lines how the start of the final adjustment phase. (cSCI = cervical Spinal Cord Injury).

Markers were placed on the right and left medial styloid process, and the distal portion of the index finger and thumb, and recorded with a 5-camera motion analysis system (Proreflex, Qualysis, Sweden) sampling at 120Hz. Data were filtered using a low-pass Butterworth filter with a cut-off frequency of 10Hz [8,30], and were then analysed using Visual3D software (C-motion, USA). Kinematic landmarks were identified on the tangential velocity profiles (see Fig 1 for examples) using a custom-written program and confirmed by concurrent visual analyses of the velocity and displacement profiles.

### Data analyses

We calculated several parameters related to the transport and grasp phases in line with past research[11,28,31–34]. From the wrist marker we computed: (1) Movement time (MT): the duration between movement onset and end, with movement onset (MO) defined as when the velocity of the wrist reached 50mm/s, and movement end (END) once the object moved in the vertical direction (z), (2) Peak velocity (PV): maximal velocity of the wrist during the reach, (3) Percentage of MT spent decelerating (PropDT): the time between PV and END expressed as a percentage of total MT, (4) Final adjustment phase (FAP): the time between the velocity of the wrist reaching 50 mm/s during the deceleration phase and END, as a percentage of total MT (PropFAP), (5) ‘Interlimb synchrony’ the absolute difference in time between the P/LI and NP/MI limbs at MO, PV, start of FAP and END.

From the markers on the thumb/s and index finger/s of each hand we calculated: (1) Maximum grasp aperture (MGA); the largest distance between the index finger and thumb during the reach, (2) the time at which this MGA occurred during MT (expressed as a percentage of total MT), (3) the coupling of the grasp and transport phase (TrG) calculated as the time of peak deceleration (time at which the wrist was decelerating fastest) minus the time of MGA, with a smaller value indicating greater coupling.

### Statistical analyses

Data were examined using separate 3 group (cSCI, YA, OA) x 2 condition (unimanual, bimanual) x 2 limb (P/LI, NP/MI) repeated measures ANOVAs. When sphericity could not be assumed F and P values were generated using the Greenhouse-Geisser correction. Significant main effects were investigated using pairwise comparisons with Bonferonni adjustments, and all significant interactions were explored using the appropriate inferential statistics. Interlimb synchrony was examined using one-way ANOVAs. Statistical significance was set at p<0.05.

## Results

### Transport phase: MT, PV, PropDT and PropFAP

**Fig 2:**
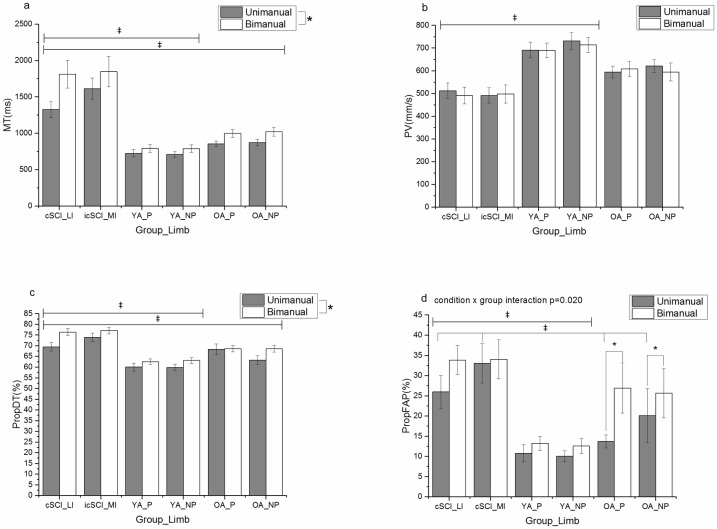
Group and limb means (±standard error) for Movement Time (MT) (a), Peak Velocity (PV) (b), proportion of movement time spent Decelerating (propDT) (c), proportion of movement time spent in Final Adjustment Phase (propFAP) (d) for unimanual (grey) and bimanual (white) conditions. (* denotes significant difference between conditions and ‡ represents a significant difference between groups), (cSCI_LI = cervical Spinal Cord Injury less impaired limb, cSCI_MI = cervical Spinal Cord Injury more impaired limb, YA_P = non-injured younger adults preferred limb, YA_NP = non-injured younger adults non-preferred limb, OA_P = non-injured older adults preferred limb, OA_NP = non-injured older adults non-preferred limb).

In comparison to the unimanual task, all participants (cSCI and UP) took longer to complete the bimanual reach-to-grasp task [F(1,46) = 7.58, p<0.01, η^2^ = 0.14] (Fig 2a). Furthermore, a longer period of deceleration (PropDT) [F(1,43) = 23.64, p<0.001, η^2^ = 0.36] and final adjustment (propFAP) [F(1,43) = 7.93, p<0.01, η^2^ = 0.16] were also noted for bimanual tasks. Fig 2a clearly shows that participants with a cSCI produced movements which were significantly longer in duration than UP (YA and OA) [F(2,46) = 27.62, p<0.001, η^2^ = 0.55] and reached a significantly lower PV than YA [F(2,43) = 9.89, p<0.001, η^2^ = 0.32] (Fig 2b). Participants with a cSCI also had a longer PropDT than UP (YA and OA) [F(2,43) = 19.94, p<0.001, η^2^ = 0.48] (Fig 2c). Differences in PropFAP [F(2,43) = 7.01, p<0.01, η^2^ = 0.25] were noted between the YA and the cSCI group with no significant difference between cSCI and OA (Fig 2d). There was no significant main effect of limb for MT, PV, PropDT or PropFAP (p>0.05).

Further analysis of the significant condition by group interaction for PropFAP [F(2,43) = 4.31, p<0.05, η^2^ = 0.17] via paired t-tests (limb collapsed) revealed that OA exhibited a longer PropFAP during the bimanual condition compared to the unimanual condition [t(14) = 3.142, p<0.01]. However, in contrast while participants with a cSCI had a longer PropFAP, this was not exacerbated by the bimanual condition; this pattern of results was also evident for the YA. One-way ANOVAs also revealed that when comparing groups, while in the bimanual condition the YA had a smaller PropFAP than participants with cSCI [F(2,46) = 6.47, p<0.01, η^2^ = 0.22] there was no significant difference between OA and people with cSCI. In contrast, in the unimanual condition differences between groups were noted between all UP and participants with cSCI [F(2,49) = 8.04, p<0.01, η^2^ = 0.25].

### Grasp phase: MGA, Time of MGA, TrG

**Fig 3:**
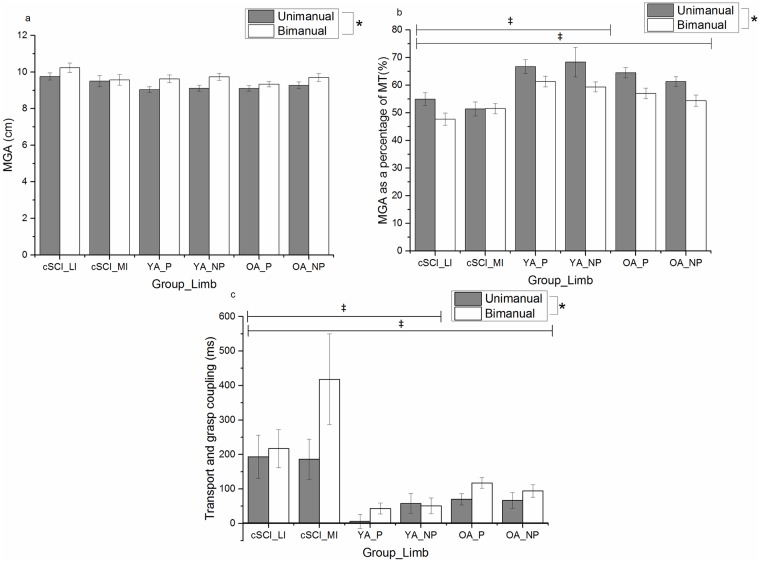
Group and limb means (±standard error) for Maximum Grasp Aperture (MGA) (a), time of Maximum Grasp Aperture as a percentage of Movement Time (MGA as a percentage of MT) (b) and transport and grasp coupling (c) for unimanual (grey) and bimanual (white) conditions. (* denotes significant difference between conditions and ‡ represents a significant difference between groups),(cSCI_LI = cervical Spinal Cord Injury less impaired limb, cSCI_MI = cervical Spinal Cord Injury more impaired limb, YA_P = non-injured younger adults preferred limb, YA_NP = non-injured younger adults non-preferred limb, OA_P = non-injured older adults preferred limb, OA_NP = non-injured older adults non-preferred limb).

While there was no difference between the groups [F(2,43) = 1.46, p>0.05, η^2^ = 0.06], the bimanual condition elicited larger MGAs than the unimanual condition [F(1,43) = 34.731, p<0.001, η^2^ = 0.447] (Fig 3a). MGA was reached earlier during the bimanual condition than the unimanual one for all participants [F(1,43) = 14.05, p<0.01, η^2^ = 0.24] and participants with a cSCI reached MGA significantly earlier than the UP [F(2,43) = 13.13, p<0.001, η^2^ = 0.38] (Fig 3b). The earlier MGA also resulted in a less coupled reach and grasp phase [F(2,43) = 15.89, p<0.001, η^2^ = 0.43] for participants with an cSCI compared to UP (Fig 3c). There was no significant main effect of limb for MGA, Time of MGA or TrG (p>0.05).

### Interlimb synchrony

**Fig 4:**
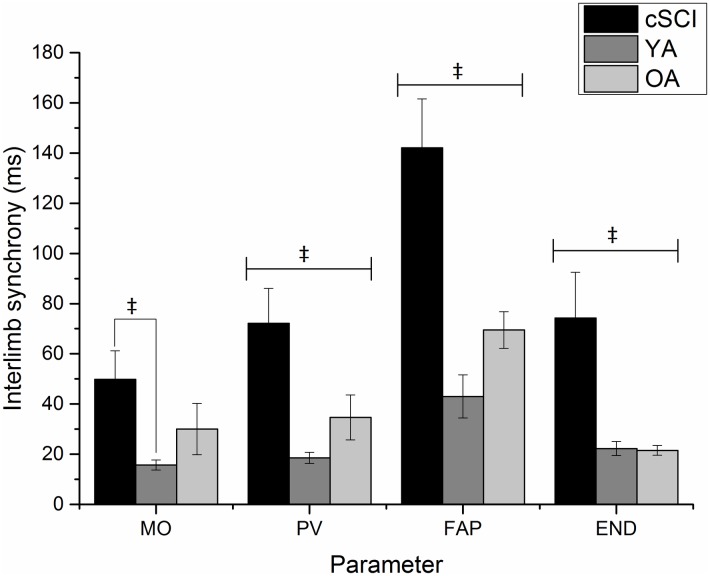
Group means (±standard error) for absolute interlimb synchrony at movement onset, peak velocity, start of the final adjustment phase and end of the movement. (‡ represents a significant difference between groups), (cSCI = cervical Spinal Cord Injury, YA = non-injured younger adults, OA = non-injured older adults).

Differences between the groups were noted for MO [F(2,46) = 3.73, p<0.05, η^2^ = 0.14], PV [F(2,46) = 7.67, p<0.01, η^2^ = 0.25], start of final adjustment phase (FAP) [F(2,46) = 14.38, p<0.001, η^2^ = 0.38], and END [F(2,46) = 6.89, p<0.01, η^2^ = 0.23] (Fig 4). Overall, analyses showed that participants with a cSCI were less synchronous than YA at the start, and less synchronous than both UP groups at each further time point. Fig 4 clearly indicates that irrespective of group the limbs become more synchronous between the start of FAP and END.

## Discussion

This paper explores control of unimanual and bimanual reach-to-grasp in individuals with an acute cSCI, and how this differs compared to uninjured younger and older adults. The main findings indicate that individuals with a cSCI produced longer, slower reach-to-grasp actions, with a longer deceleration and final adjustment phase before object pick up. While differences in kinematic characteristics were exacerbated when performing a bimanual reach-to-grasp compared to those performed unimanually (see Fig 2a–2d), both arms produced similar movement patterns (no significant main effect of limb for any of the variables). While maximal grasp aperture (MGA) was no different to uninjured participants, individuals with a cSCI reached MGA earlier in the overall reach-to-grasp. Finally, interlimb synchrony was reduced in individuals with cSCI, but endpoint temporal synchrony i.e. the point at which the object was picked up, was evident.

As expected [11–14,35,36], individuals with a cSCI produced movements of a longer duration than UP, with a lower peak velocity than YA (Fig 2a and 2b). The longer movement time was primarily due to a prolonged deceleration phase when compared to UP and final adjustment phase when compared to YA (Fig 2c and 2d). The prolonged low velocity phase suggests a more corrective mode of movement control, possibly mediated by visual feedback [28], while the overall motor slowing following cSCI, has been thought to occur in order to maintain end-point accuracy and due to declines in triceps strength [10].

When comparing unimanual and bimanual data, in general the data from uninjured adults agree with previous findings [16,17]. Bimanual movements are longer in duration with an increased reliance on the deceleration and final adjustment phase and maximal grasp aperture is larger and reached earlier [37,38]. However, this data showed no real detrimental impact of the more impaired limb on the less impaired limb as no main effects of limb or interactions emerged.

Analyses of our data showed that the increases in overall movement duration seen in individuals with a cSCI could have risen from a more prolonged deceleration phase (PropDT) and a longer final adjustment phase (PropFAP) during the reach phase (see Fig 2c and 2d). This is in contrast to previous work [5], which noted that in chronic stage of injury, arm acceleration did not differ compared to UP. This suggests that one key difference between arm function in the chronic and acute stages could be the contribution afferent feedback has in guiding transportation of the limb. Furthermore, the data suggests that visual feedback could be of even greater importance to individuals with an acute cSCI due to altered or absent afferent feedback, and this is reflected in a longer deceleration phase during arm transport (see Fig 2c). This has clinical implications as integrating somatosensory stimulation [39] or functional electrical stimulation [6] into bimanual therapy has been shown to increase cortical motor excitability and improve sensory function. This may subsequently reduce the reliance on visual feedback and therefore reduce the kinematic differences seen in acute cSCI when compared to UP.

In terms of the grasp phase, much like previous work, all participants were able to scale their MGA to the object with no group differences emerging [15]. Furthermore, MGA occurred earlier in the bimanual reach-to-grasp tasks than the unimanual one for all groups (see Fig 3b) [17]. However, participants with a cSCI produced their maximal grasp aperture significantly earlier during hand transport than UP, prolonging the time it took to close the hand around the object. This supports previous work where reaching for a small object (10m in diameter vs 30mm in diameter used in the current study) increased the time it took to close the hand after MGA [5] while reaching for a large object (75mm in diameter) caused an increase in the time it took to reach MGA [5]. The earlier MGA noted in the present study could be a compensatory mechanism to deal with the increased amount of time required to scale the hand to the object size [7] and/or the time required during hand closing to utilise visual and proprioceptive online feedback [40] to perform a successful grasping action [5].

The earlier MGA also resulted in a reduced intralimb coordination, as in UP MGA and peak deceleration are often temporally coupled [11,36], while in people with a cSCI the reach and grasp phase are performed sequentially (see Fig 3b). Other research shows that proprioceptive deficits have been found to increase the duration of hand closure [40], therefore it may be that sensory deficits of individuals with cSCI could contribute to the early MGA, and this could impact overall interlimb coordination [5].

The interlimb synchrony data shows that the people with a cSCI produced less synchronous movements than YA at movement onset and both YA and OA at all other kinematic landmarks investigated (PV, Start of FAP, END). However, as seen in Fig 4 all participants used the final adjustment phase to improve synchrony of the limbs when picking up the objects. The results of the study suggest that despite disparate abilities of the two limbs following cSCI participants still attempted to complete the bimanual task in a synchronous fashion even with no specific instructions to do so. This suggests that a level of bimanual coordination is retained and this could be improved with integration of bimanual movements into rehabilitation. Bimanual upper limb function has previously been shown to improve following bimanual therapy interventions in participants with cSCI [6, 39]. Additionally, as the participants in this study were in the acute stages of injury, incorporating bimanual therapy at this early stage will help to maximise neuroplasticity and improvements in function [41].

One potential limitation to this study is that participants had C4-C8 injuries which resulted in differing levels of upper limb muscle paresis. Although, skeletal level and time since injury were investigated as covariates no significant main effects or interactions emerged. This could have been due to the small number of participants for each skeletal level and time since injury (see Table 1). Future work could include a larger sample size split across differing skeletal levels to explore this further as research has shown that individuals with a cSCI often develop new neuromotor strategies in order to produce functional movements of the upper limbs [42]. Further work should consider using surface electromyography and examine individual joint contributions in the control of bimanual reach-to-grasp tasks to further characterise bimanual control strategies after cSCI [43]. Together, these data will enable the more targeted rehabilitation strategies where specific muscle synergies can be re/trained. Finally, the inclusion of fMRI data in future studies would allow for discussion of neurodegenerative changes following cSCI and how this may change bimanual control. This may aid the design of future bimanual therapy interventions.

The present study only examined a non-cooperative symmetrical bimanual control task. Given that interlimb coordination must be flexible to adjust to an ever-changing environment [44], and previous research has shown that altering the size of the object resulted in alterations to the movement strategy used by people with a cSCI [5], future work should examine asymmetrical reach-to-grasp tasks which pose an even more complex control problem [18], but are possibly more representative of activities of daily living.

## Conclusions

The overall clinical message from these data suggests that a level of bimanual control is retained following cSCI, and there seems to be little detrimental effect of the more impaired limb on the less impaired limb. The acute stages of the injury are known to induce the greatest neuroplasticity [41], thus incorporating bimanual therapy at this early stage may maximise functional recovery and improve bimanual upper limb function as shown by previous interventions [6, 39]. Furthermore, given what appears to be a reliance on visual/proprioceptive feedback of the limb and hand, future research should establish whether supplementing task specific practice with augmented feedback, or somatosensory stimulation could aid arm and hand recovery [6,45]. These data also show that it is important to assess bilateral impairments and quantify performance and control of bimanual tasks. Kinematic analyses of arm and hand movements are likely to provide more sensitive measures with which to judge efficacy of bimanual training strategies.

## Supporting information

S1 Table Analysis of Covariance (ANCOVA) results with skeletal level as a covariate. (DOCX)

S2 Table One way ANOVA results with skeletal level as a covariate. (DOCX)

S3 Table Analysis of Covariance (ANCOVA) results with time since injury as a covariate. (DOCX)

S4 Table One way ANOVA results with time since injury as a covariate. (DOCX)
