# Supplementary material for: Bimanual reach to grasp movements after cervical spinal cord injury
Source: PLoS One. 2017 Apr 6;12(4):e0175457. doi: 10.1371/journal.pone.0175457 (PMC5383293; doi:10.1371/journal.pone.0175457)
Supplement: S1 Table — (DOCX) [file pone.0175457.s001.docx]

| Dependant variable | Hand by level interaction | Condition by level interaction | Hand by condition by level interaction |
| --- | --- | --- | --- |
| PV | F(1,13)=0.63, p>0.05, η^2^=0.05 | F(1,13)=1.87, p>0.05, η^2^=0.13 | F(1,13)=0.42, p>0.05, η^2^=0.03 |
| MT | F(1,16)=0.65, p>0.05, η^2^=0.04 | F(1,16)=0.96, p>0.05, η^2^=0.06 | F(1,16)=1.14, p>0.05, η^2^=0.07 |
| propDT | F(1,13)=1.38, p>0.05, η^2^=0.002 | F(1,13)=3.24, p>0.05, η^2^=0.20 | F(1,13)=0.81, p>0.05, η^2^=0.06 |
| propFAP | F(1,13)=3.09, p>0.05, η^2^=0.19 | F(1,13)=8.66, p>0.05, η^2^=0.008 | F(1,13)=35.41, p>0.05, η^2^=0.03 |
| MGA | F(1,13)=0.58, p>0.05, η^2^=0.04 | F(1,13)=0.76, p>0.05, η^2^=0.06 | F(1,13)=0.016, p>0.05, η^2^=0.001 |
| MGA%MT | F(1,13)=0.003, p>0.05, η^2^=0.00 | F(1,13)=0.001, p>0.05, η^2^=0.00 | F(1,13)=0.1, p>0.05, η^2^=0.008 |
| TrG | F(1,13)=0.13, p>0.05, η^2^=0.01 | F(1,13)=0.00, p>0.05, η^2^=0.00 | F(1,13)=0.000, p>0.05, η^2^=0.00 |
| NOAA | F(1,16)=0.05, p>0.05, η^2^=0.003 | F(1,16)=2.03, p>0.05, η^2^=0.11 | F(1,13)=0.56, p>0.05, η^2^=0.03 |
| NOAF | F(1,16)=0.03, p>0.05, η^2^=0.002 | F(1,16)=0.92, p>0.05, η^2^=0.05 | F(1,16)=2.35, p>0.05, η^2^=0.13 |
