# Supplementary material for: Bimanual reach to grasp movements after cervical spinal cord injury
Source: PLoS One. 2017 Apr 6;12(4):e0175457. doi: 10.1371/journal.pone.0175457 (PMC5383293; doi:10.1371/journal.pone.0175457)
Supplement: S2 Table — (DOCX) [file pone.0175457.s002.docx]

| Dependent variable | ANOVA result |
| --- | --- |
| SYNCST | F(4,17)=0.59, p>0.05, η^2^=0.15 |
| SYNCPV | F(4,17)=0.62, p>0.05, η^2^=0.08 |
| SYNCFAP | F(4,17)=1.75, p>0.05, η^2^=0.35 |
| SYNCEND | F(4,17)=0.62, p>0.05, η^2^=0.16 |
