# Supplementary material for: Bimanual reach to grasp movements after cervical spinal cord injury
Source: PLoS One. 2017 Apr 6;12(4):e0175457. doi: 10.1371/journal.pone.0175457 (PMC5383293; doi:10.1371/journal.pone.0175457)
Supplement: S3 Table — (DOCX) [file pone.0175457.s003.docx]

| Dependant variable | Hand by time interaction | Condition by time interaction | Hand by condition by time interaction |
| --- | --- | --- | --- |
| PV | F(1,13)=0.87, p>0.05, η^2^=0.06 | F(1,13)=0.12, p>0.05, η^2^=0.03 | F(1,13)=0.00, p>0.05, η^2^=0.00 |
| MT | F(1,16)=4.32, p>0.05, η^2^=0.21 | F(1,16)=0.53, p>0.05, η^2^=0.06 | F(1,16)=2.40, p>0.05, η^2^=0.13 |
| propDT | F(1,13)=3.98, p>0.05, η^2^=0.23 | F(1,13)=0.01, p>0.05, η^2^=0.001 | F(1,13)=0.89, p>0.05, η^2^=0.06 |
| propFAP | F(1,13)=0.28, p>0.05, η^2^=0.02 | F(1,13)=0.04, p>0.05, η^2^=0.003 | F(1,13)=0.26, p>0.05, η^2^=0.02 |
| MGA | F(1,13)=0.77, p>0.05, η^2^=0.05 | F(1,13)=0.53, p>0.05, η^2^=0.04 | F(1,13)=3.47, p>0.05, η^2^=0.21 |
| MGA%MT | F(1,13)=3.19, p>0.05, η^2^=0.20 | F(1,13)=0.74, p>0.05, η^2^=0.05 | F(1,13)=2.62, p>0.05, η^2^=0.17 |
| TrG | F(1,13)=0.16 p>0.05, η^2^=0.01 | F(1,13)=2.47, p>0.05, η^2^=0.16 | F(1,13)=0.49, p>0.05, η^2^=0.04 |
| NOAA | F(1,16)=2.07, p>0.05, η^2^=0.11 | F(1,16)=2.17, p>0.05, η^2^=0.12 | F(1,13)=1.85, p>0.05, η^2^=0.23 |
| NOAF | F(1,16)=0.72, p>0.05, η^2^=0.04 | F(1,16)=0.21, p>0.05, η^2^=0.01 | F(1,16)=0.62, p>0.05, η^2^=0.04 |
