# Supplementary material for: Bimanual reach to grasp movements after cervical spinal cord injury
Source: PLoS One. 2017 Apr 6;12(4):e0175457. doi: 10.1371/journal.pone.0175457 (PMC5383293; doi:10.1371/journal.pone.0175457)
Supplement: S4 Table — (DOCX) [file pone.0175457.s004.docx]

| Dependent variable | ANOVA result |
| --- | --- |
| SYNCST | F(13,17)=2.77, p>0.05, η^2^=0.69 |
| SYNCPV | F(13,17)=2.87, p>0.05, η^2^=0.7 |
| SYNCFAP | F(1,13)=1.90, p>0.05, η^2^=0.52 |
| SYNCEND | F(13,17)=2.69, p>0.05, η^2^=0.68 |
